# Improved Facet and Edge Passivation in Near‐Infrared III‐V Colloidal Quantum Dot Photodetectors

**DOI:** 10.1002/adma.202419020

**Published:** 2025-03-23

**Authors:** Pan Xia, Sasa Wang, Yiqing Chen, Ahmet Gulsaran, Yangning Zhang, Maral Vafaie, Muhammad Imran, Amin Morteza Najarian, Yanjiang Liu, Hyeongwoo Ban, Laxmi Kishore Sagar, Mustafa Yavuz, Edward H. Sargent

**Affiliations:** ^1^ Department of Electrical and Computer Engineering University of Toronto 10 King's College Road Toronto Ontario M5S 3G4 Canada; ^2^ Mechanical and Mechatronics Engineering University of Waterloo 200 University Avenue West Waterloo Ontario N2L 3G1 Canada

**Keywords:** halides passivation, high performance, III‐V QD, InAs QD, infrared photodetector

## Abstract

Lead‐free III‐V colloidal quantum dots (CQDs) are of significant interest for their potential in near‐infrared (NIR) to short‐wave infrared (SWIR) photodetection. However, achieving effective surface passivation remains challenging, especially as larger CQD sizes introduce more complex surface facets and compositions while shifting the absorption peak from the NIR to the SWIR range. In this study, a mixed‐halide passivation strategy is developed for large InAs CQDs, an approach that led to a doubling in anti‐oxidation ability and achieves a hole mobility of 0.03 cm^2^ Vs^−1^. These in turn led to a T90 lifetime of 50 h and enhanced operating stability in photodetectors operating at 1140 nm. Density functional theory (DFT) simulations and facet characterization indicate that exposed facets and edges are well passivated using a mixture of indium halides, which provide a stronger desorption energy compared to single‐halide passivation. This approach yields photodetectors with an external quantum efficiency (EQE) of 75% and a response time of 10 ns, an advance for InAs photodetectors operating at 1140 nm.

## Graphical abstract

Increasing InAs CQD size exposes diverse facets and edges, bringing challenges for effective passivation. This work introduces a mixed‐halide passivation strategy to stabilize these facets and edges, doubling anti‐oxidation ability and boosting mobility. The resulting photodetectors achieve 75% EQE, 10 ns response time, and 50 h operational stability (10% loss) at 1140 nm, advancing InAs CQD photodetector performance.

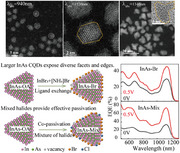

## 1 Introduction

Applying colloidal quantum dots (CQDs) in optoelectronic devices holds promise for overcoming challenges in both display and light sensing technologies. Among CQD materials, III‐V CQDs have attracted significant attention due to lead‐free composition, good thermal stability, and enhanced environmental compatibility stemming from strong covalent bonding. However, a major barrier to their widespread use is the issue of sufficient passivation, which affects not only device performance but also long‐term stability, thereby limiting their scalability in industrial applications.

Unlike II‐VI and perovskites CQDs, in which surface defects typically lead to shallow traps addressable with established passivation techniques such as ligand engineering or core–shell, III‐V CQDs exhibit deep‐level traps caused by surface defects due to their more covalent nature.^[^ ^1^, ^2^, ^3^, ^4^, ^5^ ^]^ Surface vacancies, dangling bonds, and oxides significantly affect the electronic structures of CQDs, increase defect density, and result in poor device performance and reduced stability.^[^ ^6^, ^7^ ^]^ Developing robust surface passivation strategies for III‐V CQDs is therefore of interest in order to take advantage of their prospective benefits.

A range of passivation ligands, halides,^[^ ^8^, ^9^ ^]^ sulfides, and short‐chain ligands (e.g., carboxylic acids and thiols)^[^ ^10^, ^11^ ^]^ have been found to passivate III‐V CQDs, particularly in smaller‐sized indium arsenide (InAs) CQDs. The zinc‐blende crystal structure, which dominates III‐V CQDs, leads to diverse morphologies, including tetrahedral and spherical shapes, influenced by synthesis conditions.^[^ ^12^, ^13^, ^14^, ^15^ ^]^ For example, studies show that InAs CQDs can exhibit tetrahedral or spherical shapes, depending on facet exposure during growth.^[^ ^16^, ^17^, ^18^ ^]^ These facets, however, exhibit varying trap tolerances and band structure contributions, making uniform passivation insufficient as CQD size increases. Facet‐ and edge‐specific passivation is therefore to be effective for minimizing traps, enhancing stability, and improving electronic properties.^[^ ^6^, ^19^ ^]^ Despite its importance, this approach remains underexplored, particularly for large‐size III‐V CQDs.^[^ ^20^, ^21^ ^]^

In this study, we explore the evolution of facets and edges as CQDs size increases, with InAs CQDs as our focus, investigating how the surface characteristics of zinc‐blende III‐V CQDs evolve by increasing size. Through density functional theory (DFT) simulations and detailed facet characterizations, we analyze the changes in surface characteristics−facets, edges, and vertices as size increases, aiming to guide the design of an optimal passivation strategy using halide‐based ligands. By employing mixture halides passivation strategy, we achieved InAs CQDs photodetectors with an external quantum efficiency (EQE) of 75% under 0.5 V bias and a 10 ns response time on a 2.2 mm^2^ pixel area, representing the highest EQE achieved for InAs photodetectors at 1140 nm.

**Figure 1:**
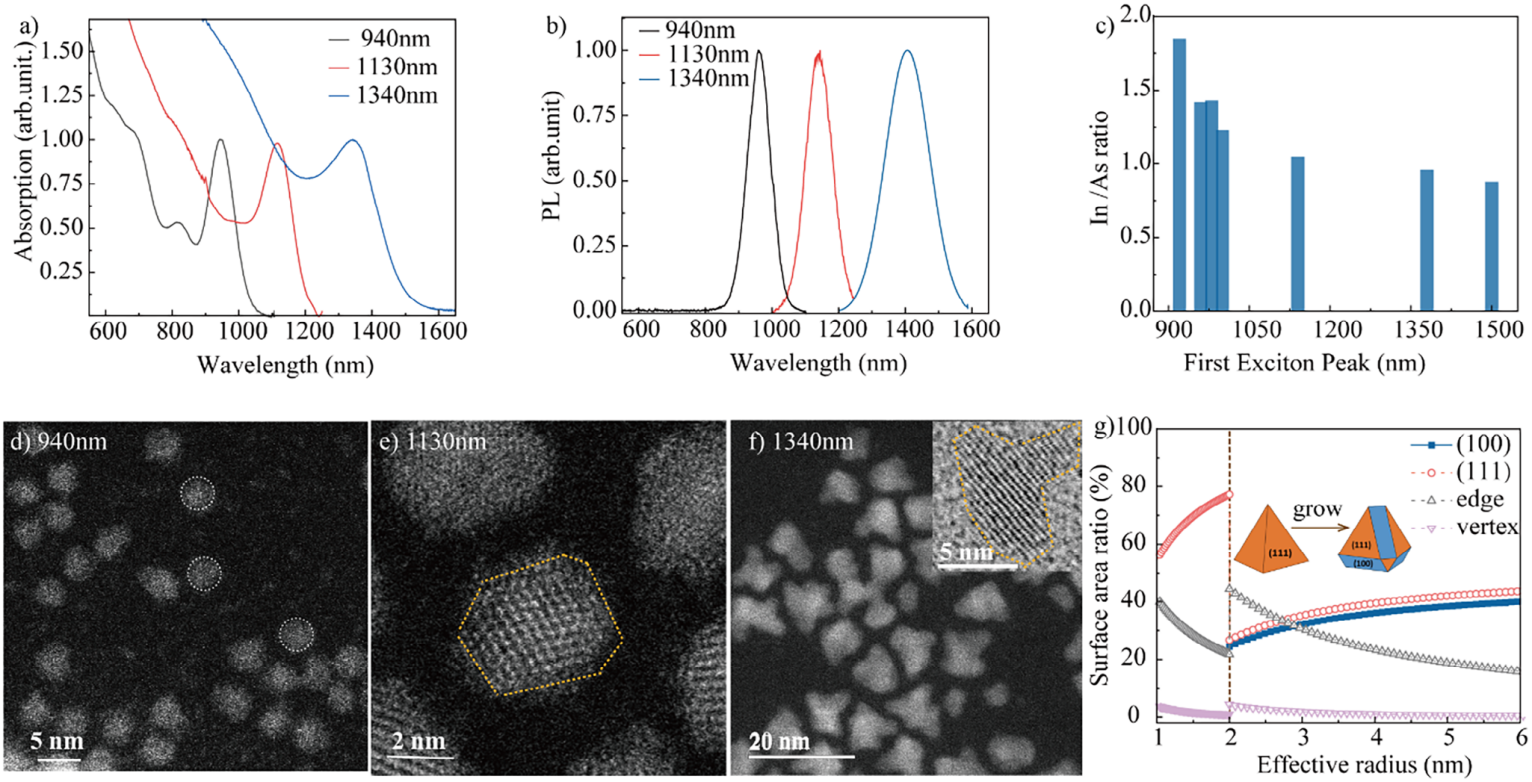
Shape and optical evolution as InAs CQDs size increases. a,b) The absorption and photoluminescence spectra of the CQDs at varying sizes. c) The ratio of indium to arsenide atoms from X‐ray photoelectron spectroscopy (XPS) measurements as the size of InAs grows, with bandgap varying from 1.4 to 0.8 eV. d–f) Transmission electron microscopy (TEM) images of the InAs CQDs, corresponding to exciton peaks ranging from 940 to 1340 nm. g) The surface area percentages of (100) and (111) facets, edges, and vertices of InAs CQDs are calculated as a function of their size. The black dashed vertical line indicates the transition point from a tetrahedral to a truncated tetrahedral shape, which is observed to occur at an effective radius of 2 nm.

We explored the facet and edge evolution of InAs CQDs synthesized using the hot‐injection method, producing exciton peaks ranging from 940 to 1340 nm.^[^ ^17^, ^22^ ^]^ The absorption and photoluminescence (PL) spectra of the CQDs are shown in **Figure** **1**a,b. Morphology was characterized using transmission electron microscopy (TEM), as shown in Figure 1d–f, revealing that the sizes increased from 1.8 to 5.8 nm, corresponding to exciton redshifts in absorption. According to the literature, high energy (100) facets limit the potential shapes of InAs CQDs, favoring cuboctahedra or In rich, (111) terminated tetrahedra at small sizes.^[^ ^12^ ^]^ As the CQDs grow, additional (100) and (110) facets emerge alongside the existing (111) facets, driving the shape transition toward polyhedral forms (Figures S1–S3, Supporting Information). For CQDs absorbing at 940 nm, their small size makes clear recognition these facets difficult, consistent with previous reports for InAs and In(As,P) CQDs.^[^ ^13^, ^18^, ^22^ ^]^ Future growth is expected to stabilize these emerging polyhedral shapes as the ratio of different facets reaches equilibrium. The stable (111) facets observed in III‐V CQDs like InP and InAs could eventually merge with new (100) and (110) facets as the CQDs increase in size. Additional TEM images and X‐ray diffraction (XRD) data supporting this shape evolution are available in Figures S1–S4 (Supporting Information). The exposure of (100) and (110) facets leading to more pronounced inhomogeneities, necessitating the passivation of these newly exposed facets, along with the edges and junctions at facet joints, to achieve high‐performance III‐V CQD photodetectors.

Figure 1c shows the indium‐to‐arsenide ratio obtained from X‐ray photoelectron spectroscopy (XPS) analysis of CQD thin films, revealing a decrease in this ratio with increasing size, indicating In‐rich InAs, especially in small size. To design an effective passivation strategy, the evolution of surface facet with increasing CQD sizes was analyzed using CQD models constructed via Wulff construction (Figure 1f).^[^ ^23^, ^24^ ^]^ Consistent with experimental observations, the (100) facet was introduced when the CQD radius reaches 2.0 nm. We calculated the changes in the (111) and (100) facet ratios as the radius expanded from 1 to 6 nm. The results revealed that the (100)/(111) ratio approached 1:1 as the size increased, indicating a stabilization in the relative proportions of these facets.

Previous studies on PbS and InP CQDs show that different facets respond differently to passivation.^[^ ^1^, ^25^ ^]^ The polar (111) facet is more easily passivated, while the charge neutral (100) facet requires additional efforts despite its increasing contributions to electronic states.^[^ ^19^, ^26^ ^]^ In III‐V CQDs, where the thermally stable shape is tetrahedron‐based, research has primarily focused on the growth and passivation of (111) facets, while (100) facets and the joint passivation of facets and edges remain less explored. Edges and vertices, being less coordinated and more exposed than facets, are highly reactive and significantly influence the electronic properties of CQDs. Additionally, studies have shown that voids can form at the edges of perovskite nanocrystals,^[^ ^27^ ^]^ highlighting the importance of a comprehensive passivation strategy that addresses facets, edges, and vertices.

**Figure 2:**
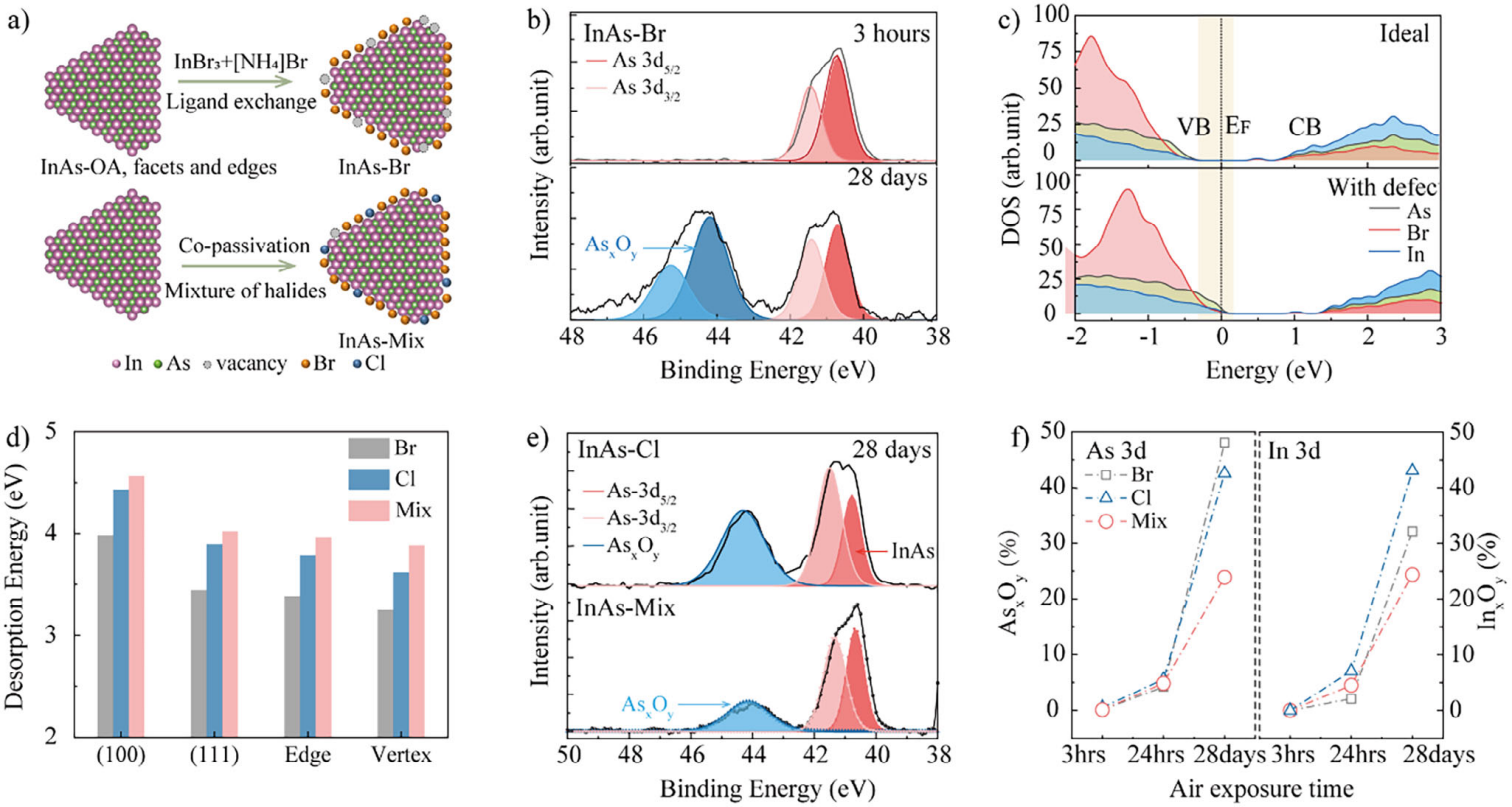
Characterization of Br‐passivated InAs CQDs and photodetectors. a) Schematic illustration of ligand passivation for large size InAs CQDs compared to single Br‐ ligand exchange process. b) XPS spectra of As 3d for Br‐passivated CQDs, analyzed after 3 h versus 28 days of air exposure. c) Density of states (DOS) of the Br‐passivated InAs CQD with ideal passivation (top) and with one exposed vertex (bottom). d) Desorption energy (E_desorp_ = E(In_136_As_96_X_119_)‐ E(In_136_As_96_X_120_)+E(X)) for the removal of a single halide atom from the (100) and (111) facets, edges, and vertices of In_136_As_96_X_120_. For the mixture halides system, the desorption energy is calculated by removing the Cl atom from In_136_As_96_Br_119_Cl. e) Experimental XPS spectra of As 3d for InAs‐Cl and InAs‐Mix films after 28 days of air exposure. f) The oxidation percentages of As (left) and In in InAs‐ligand films upon air exposure were extracted from experimental XPS analysis.

The as‐synthesized CQDs with their original long aliphatic chain ligand, oleic acid (OA), require ligand replacement to enhance CQD film conductivity for photodetector applications. To initiate this research, we applied previously reported ligand exchange methods for InAs CQDs, using InBr₃ and [NH₄]Br to replace OA, as illustrated in **Figure** **2**a.^[^ ^8^, ^28^ ^]^ We aim to explore the limitations of the current single halides passivation strategy by integrating DFT simulations with experimental data. This study focuses on the InAs CQDs with an absorption exciton at 1140 nm, a wavelength range where facets, edges and vertices are begun to be observable. The long‐term stability of Br‐passivated InAs CQDs (InAs‐Br) under ambient conditions was evaluated, using oxidation rate as a key indicator of passivation quality. After 24 h of exposure to air, 5.4% of the surface converted to indium oxides (In_x_O_y_), and 10% to arsenic oxides (As_x_O_y_) (see Figures S5 and S6, Supporting Information). Over time, the oxidation continued to progress. After 28 days of exposure, 30.3% of the surface had converted to In_x_O_y_, and 49.1% to As_x_O_y_, as shown in Figure 2b.

This oxidation results from incomplete passivation, where reactive surface dangling bonds interact with oxygen and moisture, forming oxide states and defect traps. Density of states (DOS) calculations reveal that passivation loss at the vertex site shifts electronic states and the valence band closer to the Fermi level, introducing states at the Fermi energy and resulting in heavily p‐doped InAs CQDs, as shown in Figure 2c and Figure S7 (Supporting Information).^[^ ^6^, ^29^ ^]^

To explore the impact of different passivation strategies on CQDs stability, we used DFT simulations to evaluate the desorption energies of various halides (e.g., Cl^−^, Br^−^, and a 1:1 mixture of Cl^−^ and Br^−^) on multiple CQD facets, including the (111) and (100) surfaces, as well as edge and vertex positions. This cluster model is based on previous works.^[^ ^30^, ^31^ ^]^ The results indicate that chloride (Cl^−^) and mixture halides exhibit stronger desorption energies than bromide (Br^−^), particularly on the (100) and (111) facets (Figure 2d), providing better surface stabilization and reducing surface reactivity. Similar trends were observed for edge and vertex positions using cluster models. The enhanced desorption energy of the halide mixture, compared to individual Cl⁻ or Br⁻, may be attributed to the varying desorption energies of facets other than (111) and (100) when interacting with Br⁻ and Cl⁻. This is consistent with observations that halides have different binding energies on PbS and PbSe CQD facets, as well as on metal nanostructures facets.^[^ ^2^, ^32^, ^33^ ^]^ Further investigation is needed to fully understand the passivation mechanisms of InAs CQD facets, particularly the interactions of hybrid indium halides (Z‐type ligand) and ammonium halides (X‐type ligand) with different facets.

Noting these DFT findings, we evaluated the anti‐oxidation performance of Cl‐passivated and mixture halides‐passivated InAs CQDs. Mixture halides showed better stability, with only 24.4% In_x_O_y_ and 23.8% As_x_O_y_ formed after 28 days (Figure 2e,f; Figures S5 and S6, Supporting Information). In contrast, Cl‐passivated CQDs showed rapid degradation, reaching 42.6% In_x_O_y_ and 43.1% As_x_O_y_ after the same period (Figure 2e,f). Notably, mixed halides passivation improved arsenic oxidation resistance by 2.9× compared to Br‐passivated CQDs. This enhancement can be accounted for two mechanisms: direct passivation of arsenide; and the protection of indium, this latter slowing oxygen diffusion and further shielding arsenic from oxidation.

**Figure 3:**
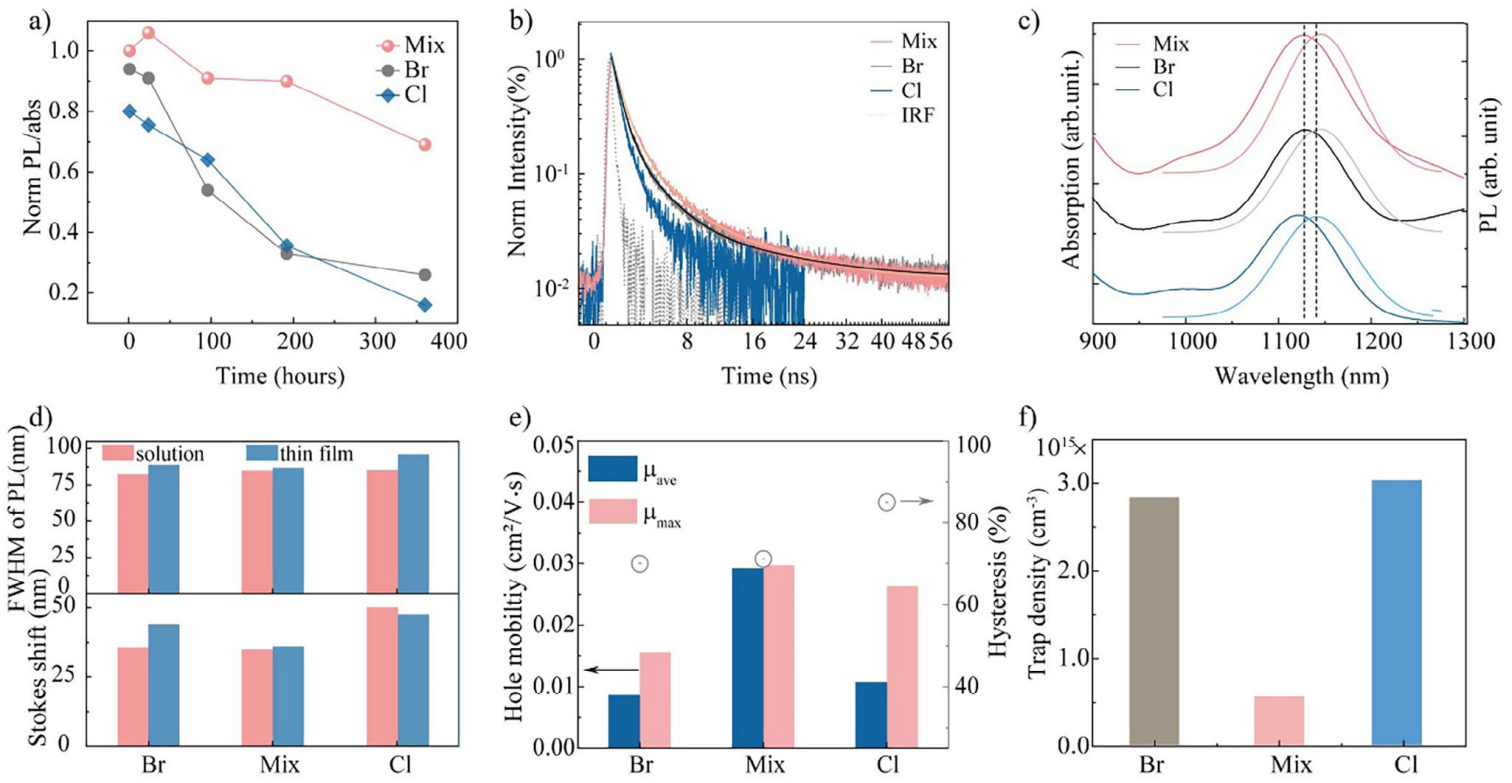
Optical characterization of InAs CQDs with different passivation. a) Ink stability of the ligand exchanged‐CQDs overtime, ranging from 1 to 350 h; b) Time‐corelated photoluminescence (PL) of InAs‐ligands CQDs in solution. The solid lines represent the fits, with details provided in Table S2 (Supporting Information); c) Absoption and PL of CQD films on glass substrates; d) PL full width at half maximum (FWHM, top) and Stokes shift (bottom) of InAs CQDs in solution and as films; e) Mobility and hysteresis of the InAs CQDs passivated with different halides, determined from the filed effect transistor (FET) structure measurements; f) Trap density of the InAs CQD films with different halide passivations, calculated by space‐charge‐limited current (SCLC) measurements.

We assessed the optical and carrier properties of the 1140 nm‐absorbed InAs CQDs with different passivation strategies. Ink stability was studied through absorption and PL measurements, with absorption spectra showing no significant changes (Figure S8, Supporting Information), indicating good colloidal stability.^[^ ^8^ ^]^ **Figure** **3**a shows that, after 360 h of storage under nitrogen, the relative PL intensity of InAs CQDs ink with mixture halides passivation retained 69% of its initial value, compared to only 26% retention for CQDs passivated individually with Br^−^ or Cl^−^. Time‐correlated PL measurements reveal a slightly longer lifetime for the mixture halides passivation as compared to the pure halide passivation (Figure 3b; Table S2, Supporting Information). Additionally, thin‐film PL data (Figure 3c,d) indicated a narrower full width at half maximum (FWHM) with mixture halides passivation, possibly due to decreased trap states.

Building on these optical stability improvements, field‐effect thin film transistors were fabricated using CQDs with different passivation strategies to measure carrier mobility. The resulting hole mobilities for Mix, Br, and Cl passivation were 0.03 ± 0.009, 0.008 ± 0.001, and 0.011 ± 0.01 cm^2^ Vs^−1^, respectively. Notably, InAs‐Mix showed the lowest hysteresis (Figure 3e; Figure S9, Supporting Information), further corroborating the enhanced passivation observed. To gain deeper insights, we studied the trap density in halide‐passivated InAs CQD films using hole‐only space‐charge limited current (SCLC) measurements. The results showed that trap density decreased significantly from 3.2 × 10^16^cm^−^^3^ for Br‐passivation to 5.0 × 10^15^ cm⁻^3^ with mixed halide passivation (Figure 3f; Figure S10, Supporting Information). This improvement in carrier mobility and reduction in trap density are attributed to the passivation of surface traps.

**Figure 4:**
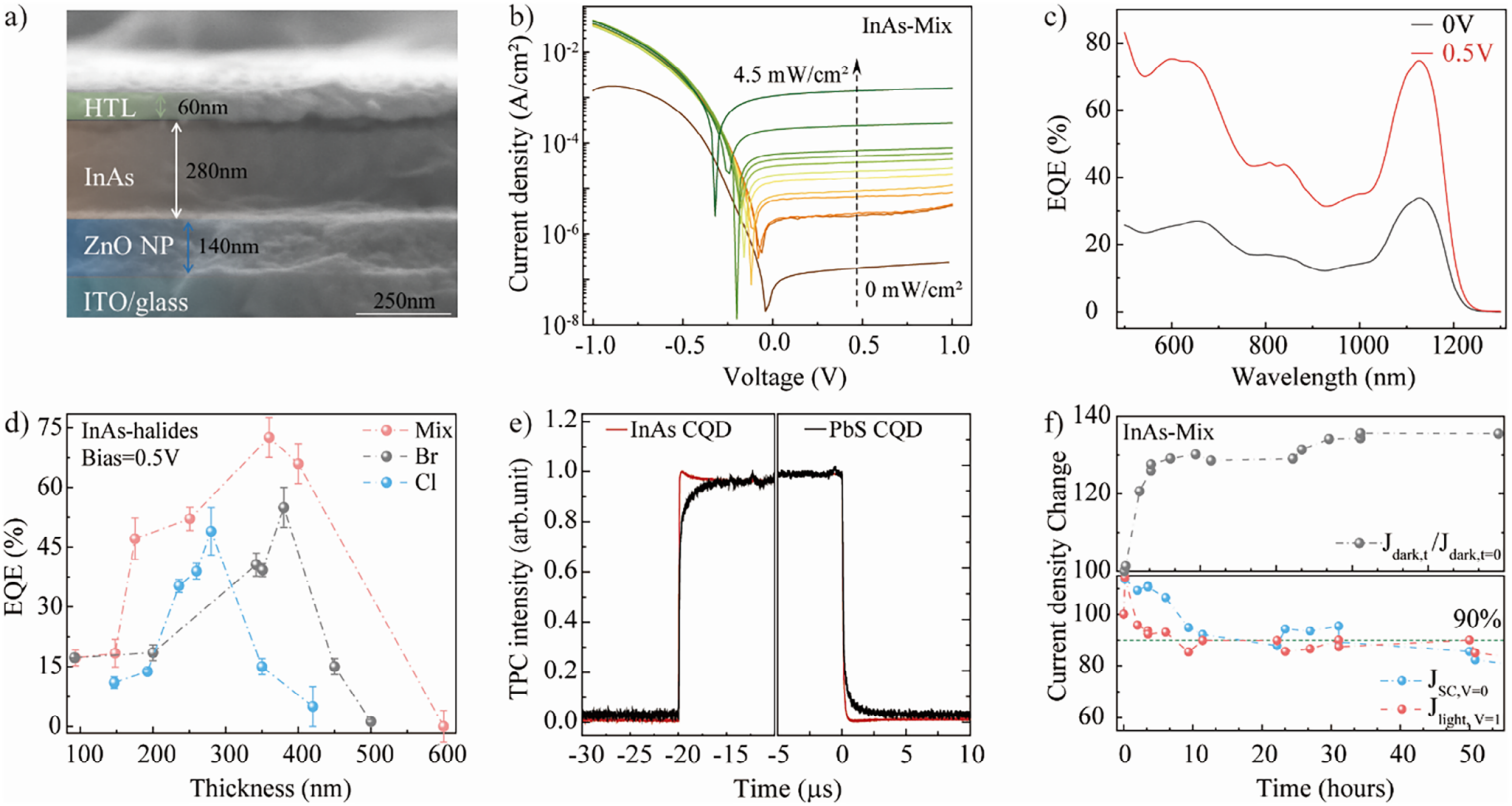
Performance of InAs‐mixed‐ligand‐based photodetectors. a) SEM cross‐section of the a typical InAs‐Mix CQD‐based photodetectors; b) Light power dependence of *J*–*V* curves of mixture halides‐passivated InAs CQDs with 940 nm excitation; c) Otpimzied EQE of the photodetectors without bias and under 0.5 V bias; d) the otpimzied EQE of the photodetectors without bias and under 0.5 V bias; d) Thickness‐dependent EQE of InAs photodetectors with different halide passivations under 0.5 voltage bias; e) Light response time of the InAs and PbS CQDs device under 850 nm exciation with 2.2 mm^2^ pixel size; f) Dark and light current stability of the InAs‐mixture CQD‐based photodetectors under illumination.

We further fabricated photodetectors using the above‐mentioned halide‐passivated InAs CQDs absorbing at 1140 nm, incorporating ZnO nanoparticles as the electron transport layer and MoO_x_/CQD as the hole transport layer (**Figure** **4**a). By optimizing the film thickness to 320 nm and the Br‐to‐Cl ratio to 1:1 (Figure S11, Supporting Information), we achieved devices with dark current of 0.1 µA cm^−^^2^ at 0.5 V and 0.24 µA cm^−2^ at 1 V (for a 10 mm^2^ pixel size, Figure 4b). Additionally, we investigate the light power dependence, highlighting the photodetector's potential for 1140 nm infrared detection applications. Using the mixture halides‐passivated CQDs, the photodetectors achieve external quantum efficiency (EQE) of 40 ± 4% without bias and 75 ± 3% at 0.5 V (Figure 4c) with. These EQE values and dark current are largely improved compared to the Cl‐ and Br‐passivated devices (Figure 4d), with a 36% improvement in EQE and 10× reduction in dark current compared to InAs‐Br‐based photodetector (Figure S12, Supporting Information), consistent with the enhanced optical and carrier properties from the previous analysis. All reported EQE are from the optimal thickness for each CQDs with the thickness dependence investigation (Figure 4d). It is worth noting that this is the highest reported EQE at 0.5 V for SWIR III‐V CQD‐based photodetectors.^[^ ^11^, ^28^, ^34^ ^]^ We also investigated the photodetectors with 940 nm‐absorbing CQDs using different ratios of Cl‐ and Br‐, finding no improvement from mixed halides compared to Br alone (Figure S13, Supporting Information).

We measured the response time, a key parameter for photodetectors, using a 2.2 mm^2^ pixel size and a continuous laser triggered by a 50 µs square pulse, achieving a 10 ns response (Figure 4e), —the fastest reported to date without femtosecond lasers. Additionally, device stability was assessed, showing a T90 lifetime of 50 h under continuous 940 nm excitation, 4.0 mW cm^−^^2^ continuous light exposure demonstrating the robust passivation and longevity of the mixture halides strategy. In comparison, InAs‐Br‐based photodetectors exhibited slightly better dark current stability but shorter T90 lifetimes, with 20 h for J_SC_ at 0 volts and 30 h for light current at 1 volt (Figure S14, Supporting Information).

## 2 Conclusion

In conclusion, we addressed the challenges of facet exposure and surface passivation in SWIR‐range InAs CQDs using a mixture‐halides passivation strategy, particularly targeting vulnerable surface arsenic atoms. This approach enhanced photodetector performance, achieving an EQE of 75 ± 3% at 0.5 V—a 1.36x increase compared to single halide passivation—and enabled a fast 10 ns light response under nanosecond laser illumination. Encapsulated photodetectors maintained 90% of their initial performance after 50 h of continuous laser operation. These results demonstrate the potential of InAs CQDs to reach EQE levels comparable to PbS CQDs and suggest a promising pathway for improving passivation in larger III‐V CQDs designed for longer‐wavelength absorption.

## Conflict of Interest

The authors declare no conflict of interest.

## Supporting information



Supporting Information

## Data Availability

The data that support the findings of this study are available in the supplementary material of this article.
