# Supplementary material for: Improved Facet and Edge Passivation in Near‐Infrared III‐V Colloidal Quantum Dot Photodetectors
Source: Adv Mater. 2025 Mar 23;37(18):2419020. doi: 10.1002/adma.202419020 (PMC12051821; doi:10.1002/adma.202419020)
Supplement: Supplementary file 1 — Supporting Information [file ADMA-37-2419020-s001.pdf]

ADVANCED MATERIALS

Supporting Information

for *Adv. Mater.*, DOI 10.1002/adma.202419020

Improved Facet and Edge Passivation in Near-Infrared III-V Colloidal Quantum Dot Photodetectors

*Pan Xia, Sasa Wang, Yiqing Chen, Ahmet Gulsaran, Yangning Zhang, Maral Vafaie, Muhammad Imran, Amin Morteza Najarian, Yanjiang Liu, Hyeongwoo Ban, Laxmi Kishore Sagar, Mustafa Yavuz and Edward H. Sargent**

Improved Facet and Edge Surface Passivation for Near-Infrared III-V Colloidal Quantum Dot Photodetectors

Pan Xia[1,†], Sasa Wang[1,†], Yiqing Chen[1,†], Ahmet Gulsaran[2], Yangning Zhang[1], Maral Vafaie[1], Muhammad Imran[1], Amin Morteza Najarian[1], Yanjing Liu[1], Hyeongwoo Ban[1], Laxmi Kishore Sagar[1], Mustafa Yavuz[2], Edward H. Sargent[1,*]

Contents

Figures 2
Experimental sections 12
- Computational methods 12
- Colloidal Quantum Dot Synthesis 12
- Colloidal Quantum Dot Photodiode Fabrication (NIP) 13
- Colloidal Quantum Dot Photodiode Fabrication (PIN) 13
- Current-voltage and External Quantum Efficiency Measurement 13
- TPC and photodetector stability measurement 13
- Noise-equivalent power measurement and specific detectivity calculation 13
- Absorption, PL and TRPL measurement 14
- FET fabrication and measurement 14
- Space-charge-limited current (SCLC) measurement 14
- X-ray Photoelectron Spectroscopy (XPS) 14
- Ultraviolet photoelectron spectroscopy (UPS) measurement 15
- Transmission Electron Microscopy (TEM) Characterization 15
- Powder X-ray scattering diffraction (PXRD) 15

Figures

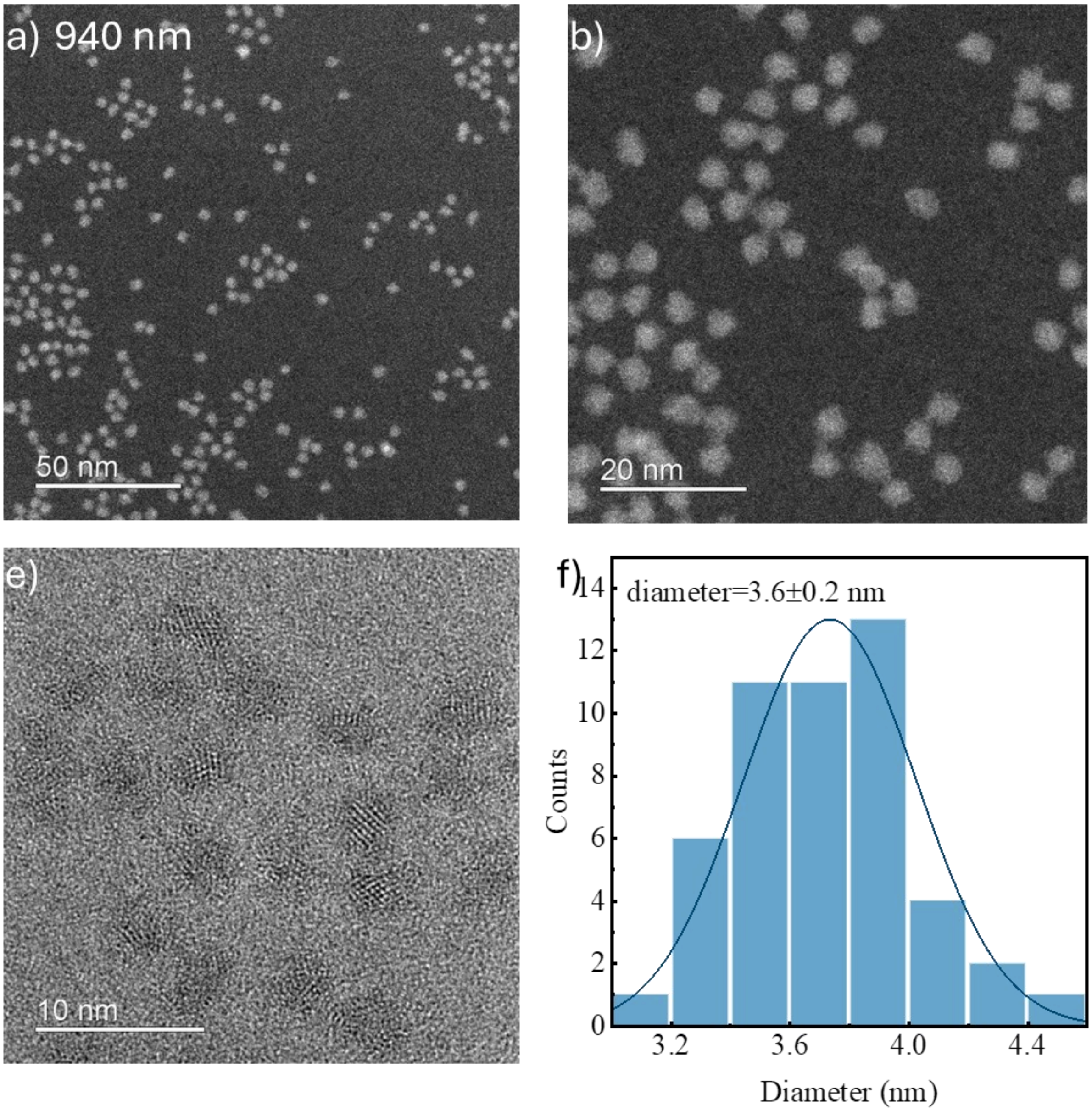

Figure S1. TEM images of InAs CQD absorbing 940 nm (a-c), and the size distribution(d). The scale bars are in figures.

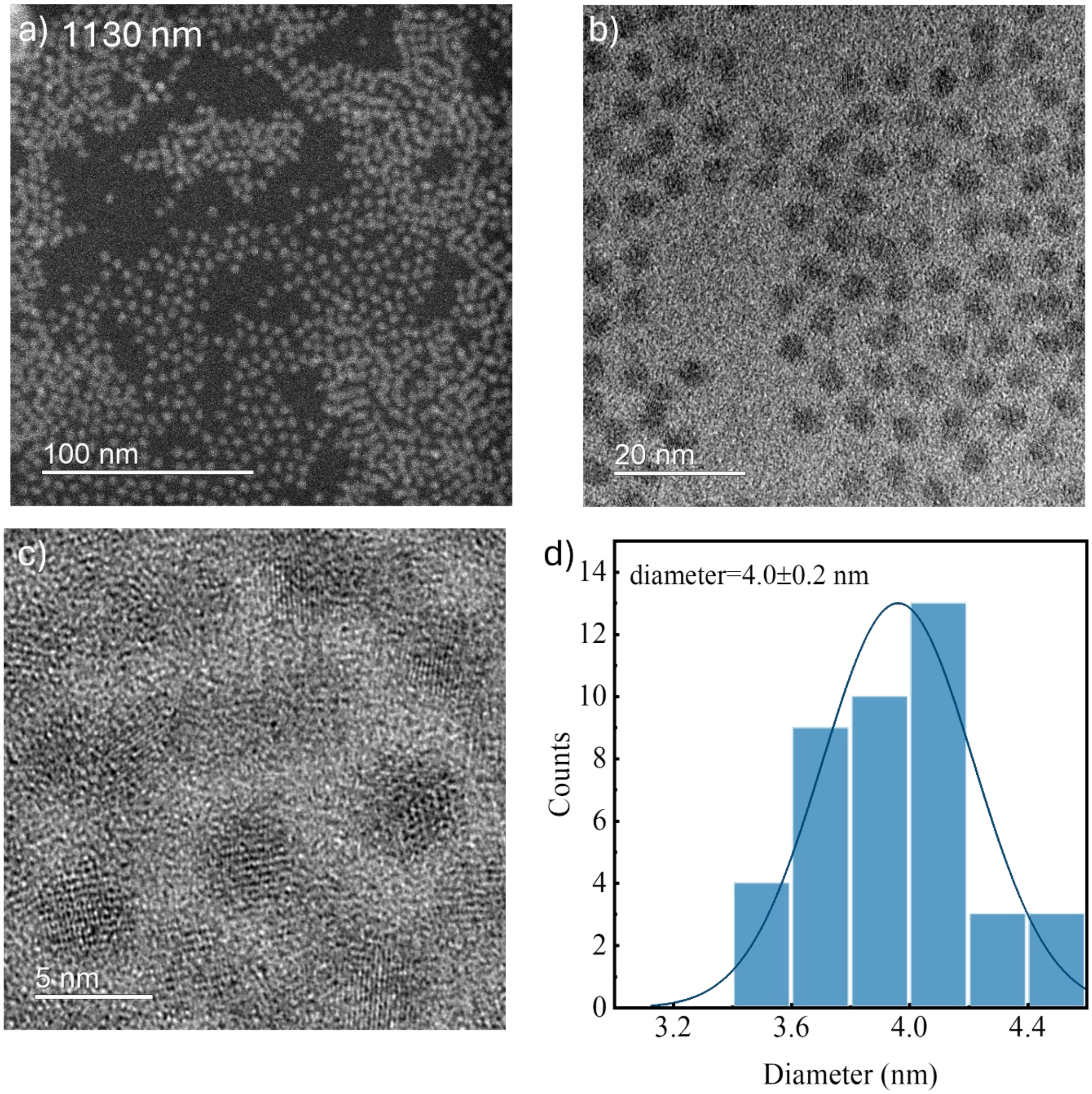

Figure S2. TEM images of InAs CQDs absorb at1130 nm (a-c), and the size distribution(d). The scale bars are in figures.

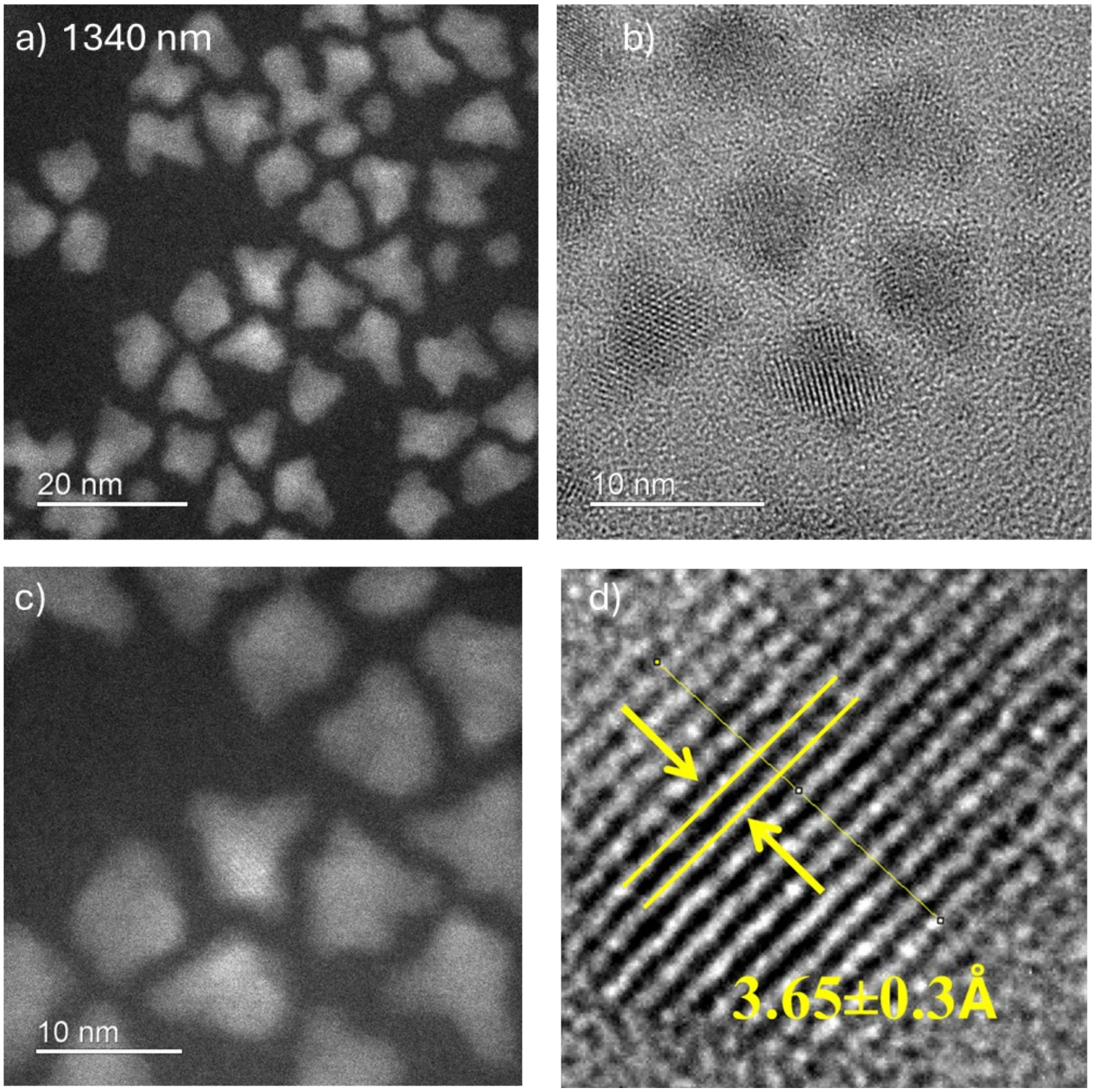

Figure S3. TEM images of InAs CQD absorbing 1340 nm (a-c), and the lattice spacing(d). The scale bars are in figures.

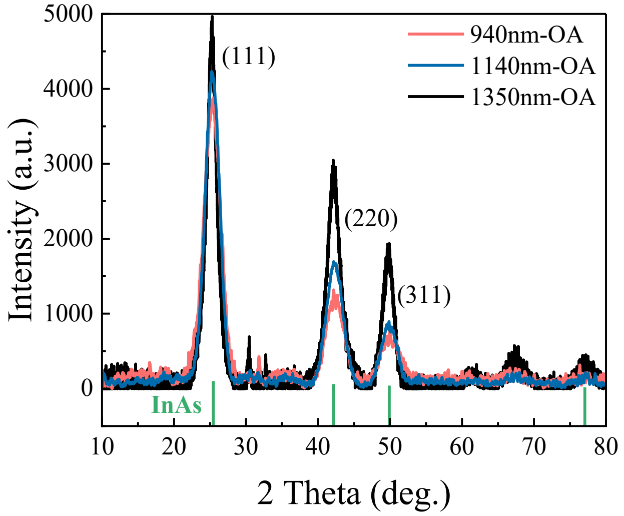

Figure S4. XRD patterns of InAs CQDs at different sizes. The relative peak intensity (I_{220}/I_{111}) of the (220) and (111) facet is calculated as 0.12, 0.23 and 0.61 for the 940, 1140 and 1350 nm absorbed films, indicating the increased (220)-facet exposure as the size increase in CQDs.

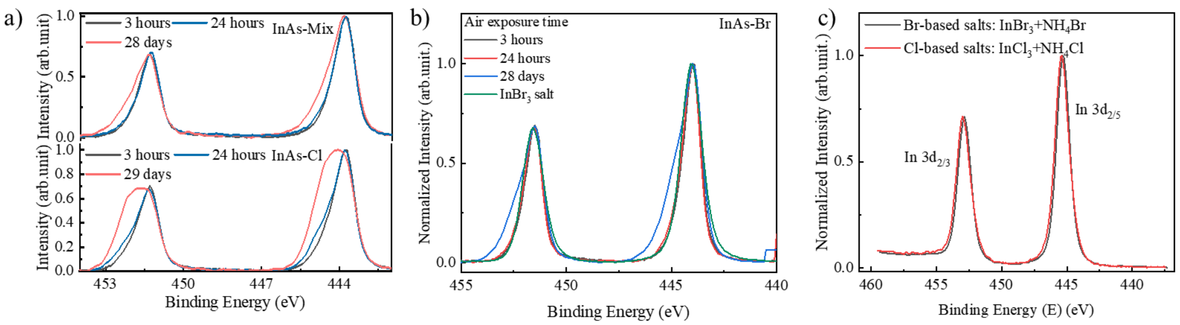

Figure S5. XPS of Indium 3d. a) of different InAs CQD absorbing at 1140 nm samples with Br, Cl and Mix passivation after 3 hours, 24 hours, and 28 days of air exposure. b) of InAs CQD with Br passivation after 3 hours, 24 hours, 28 days, and $InBr_3$ salt (powder). c) of different salts. We believe that it's difficult to separate the In-As and In-halides (Br and Cl) peaks here.

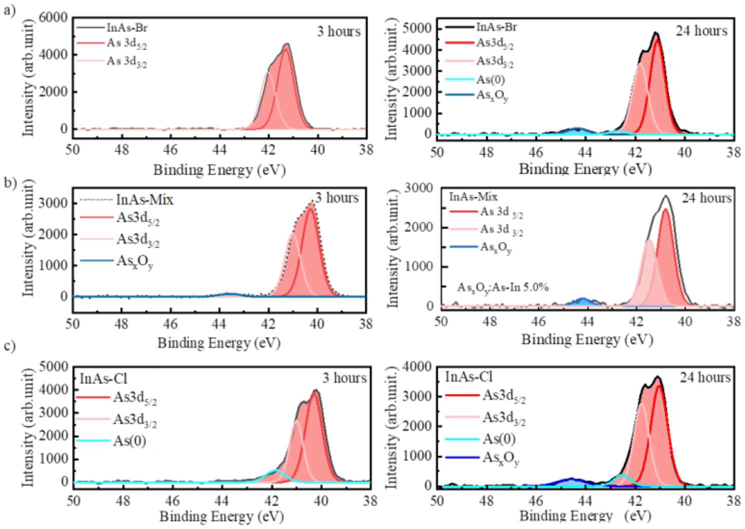

Figure S6. XPS of arsenide of different InAs CQs absorbing at 1140 nm with Br, Mix and Cl as ligands after 3 hours and 24 hours air exposure.

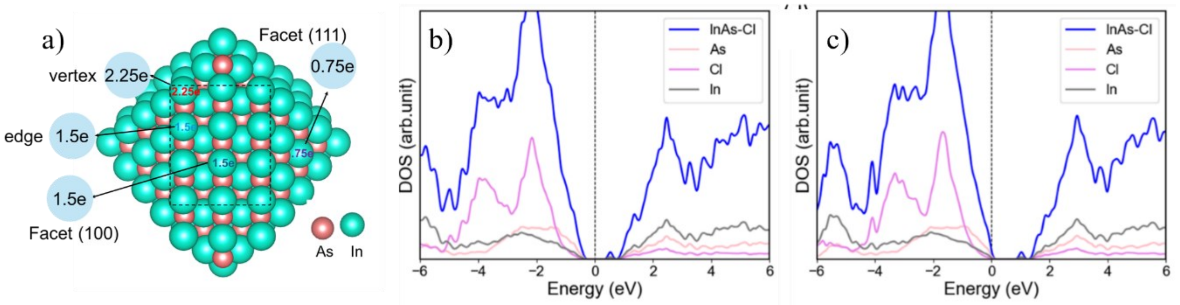

Figure S7. Schematic representation of an InAs QD ($In_{136}As_{96}$). The numbers indicate the number of dangling bonds per surface indium atom on the (100) facets (blue), (111) facets (purple), edges (yellow), and vertices (red). Total and projected density of states (PDOS) for (a) fully Cl-passivated QD, and (b) Cl-passivated QD with one exposed vertex. The Fermi level is set to 0 eV.

Compared to the In atoms on the more stable (111) facets, those located at the edges and vertices are under-coordinated, resulting in a higher number of dangling bonds, which increases surface energy and contributes to their instability. While the In atoms on the (100) facets have a similar number of dangling bonds as those at the edges, the atoms at the edges are more exposed to the environment, increasing their reactivity and making them higher-energy sites.

Table S1. Oxides ratio increase as air exposure time. Here the ratio is the oxides to the InAs bonds from XPS.

	3hrs	24hrs	28days	3hrs	24hrs	28days
Elements	In 3d	In 3d	In 3d	As 3d	As 3d	As 3d
Br	0%	2.1%	32.2%	0.10%	4.21%	49.08%
Cl	0%	7.1%	43.1%	0.50%	5.57%	42.56%
Mix	0%	5.2%	24.4%	0.10%	4.85%	23.90%

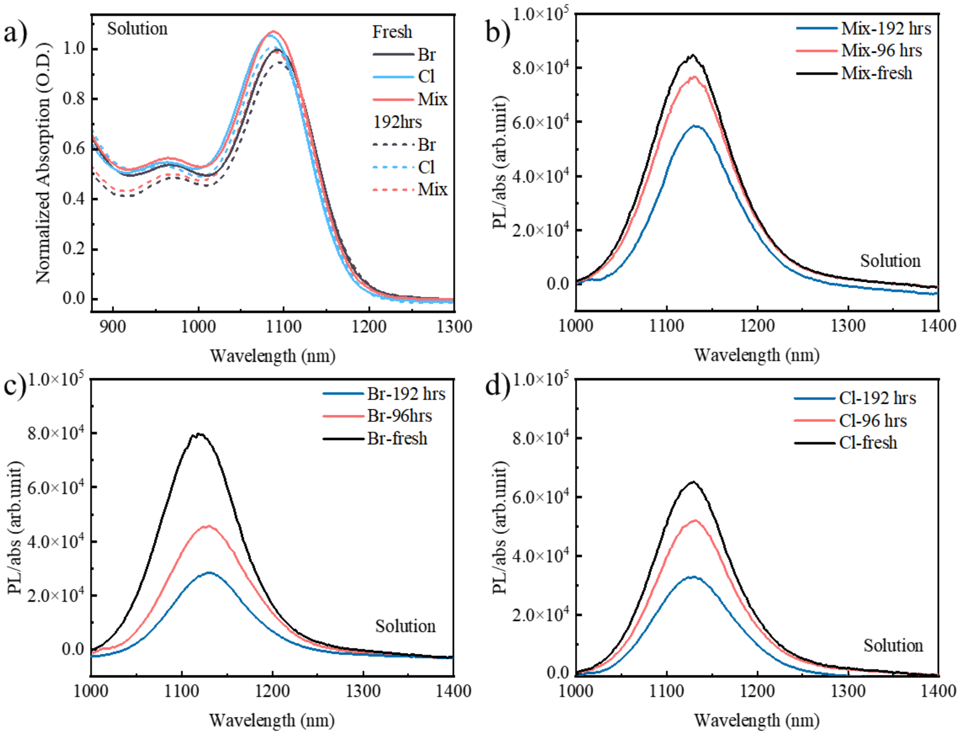

Figure S8. Absorption and photoluminescence of InAs (abs=1100 nm) after ligand exchange normalized by absorption at 24 hours, 192 hours and 360 hours. The ink is relatively stable under nitrogen, as it shows less than 0.5% changes in absorption intensity.

Table S2. Fitting parameters for TRPL of InAs with Mix-, Br- and Cl- passivation.

	Mix		Br		Cl	
	Value	Std error	Value	Std error	Value	Std error
A1	0.72	8E-03	0.19	3.62E-04	1.03	9.48E-04
t1	0.73	7E-03	2.85	0.00E+00	0.70	0.00E+00
A2	0.26	7E-03	0.86	6.14E-03	0.08	8.65E-03
t2	2.45	7E-02	0.70	1.35E-01	4.18	1.02E-02
A3	0.05	3E-03	0.02	7.72E-03	-	-
t3	11.27	5E-01	14.49	6.53E-03	-	-
Amplitude lifetime $\sum Ai * t_i / \sum A_i$	1.65	0.11	1.40	0.42	0.96	0.23
Weighted lifetime $\sum A_i^2 * t_i // \sum A_i^2$	4.65	0.38	4.78	1.67	1.82	0.44

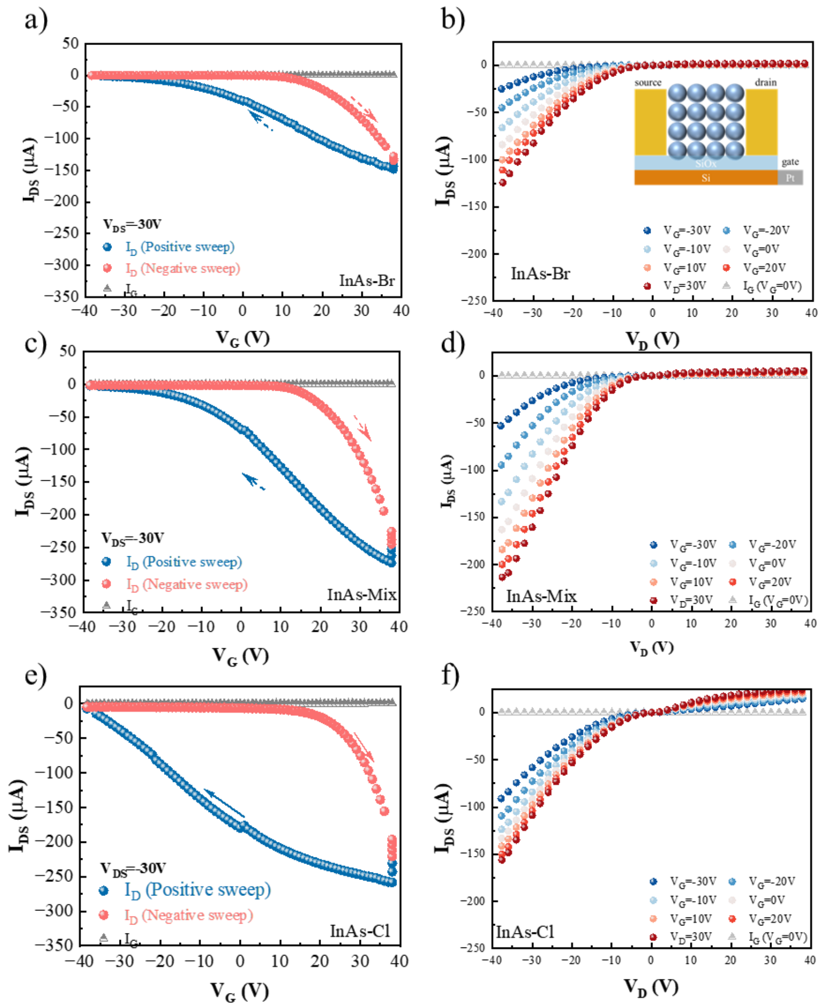

Figure S9. InAs-halides FET. InAs CQD film transfer curves with various ligands, a) Br, c) Mix, e) Cl, and corresponding output curves, b) Br, d) Mix, and f) Cl. The FET structure diagram is shown in the inset.

Table S3. SCLC calculation parameters

	**Parameters**	**Mix**	**Cl**	**Br**
**Parameters**	V_{tfl} (V)	0.214	1.17	1.09
**dielectric const**	ε	14.6	14.6	14.6
**electron charge**	e (C)	$1.6*10^{-19}$	$1.6E*10^{19}$	$1.6*10^{-19}$
**length**	L (nm)	250	250	250
**trap density**	N_t (cm^{-3})	$5.53*10^{15}$	$3.02*10^{16}$	$2.82*10^{16}$
SCLC regime				
**current density**	J (A/μm^2)	585	232	663
**voltage**	V (V)	3.52	4.13	5.17
**dielectric const**	ε	14.6	14.6	14.6
**length**	L (nm)	250	250	250
**mobility**	μ (cm^2/Vs)	$1.04*10^{-3}$	$2.98*10^{-4}$	$5.44*10^{-4}$

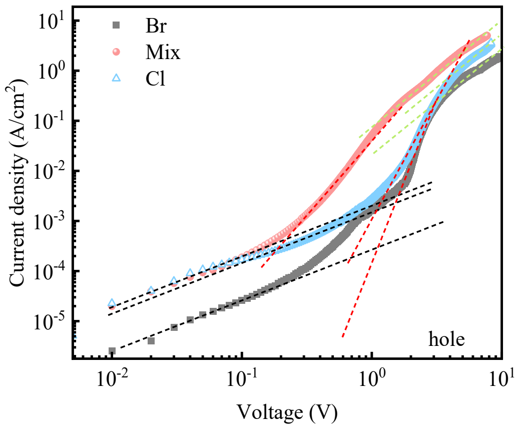

Figure S10. Hole-only space charge limited current (SCLC) measurements of quantum dot (QD) devices. The device structure is ITO/PTAA (10nm)/InAs(250nm)/MoOx(14nm)/Ag.

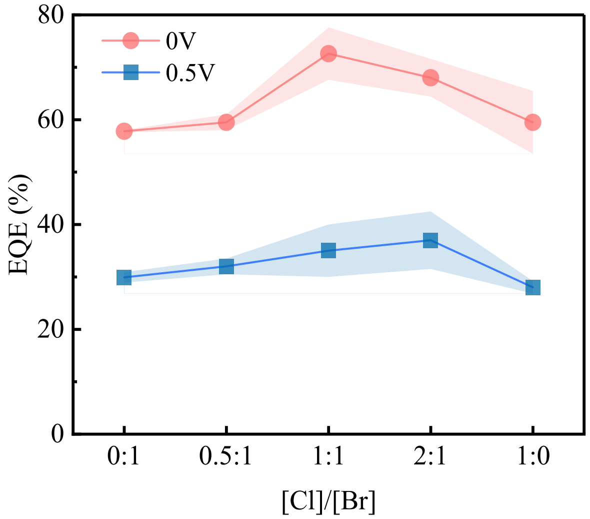

Figure S11. Dependence of EQE of InAs QD photodetector on the Cl-to-Br ([Cl]/[Br]) ratio in ligand exchange.

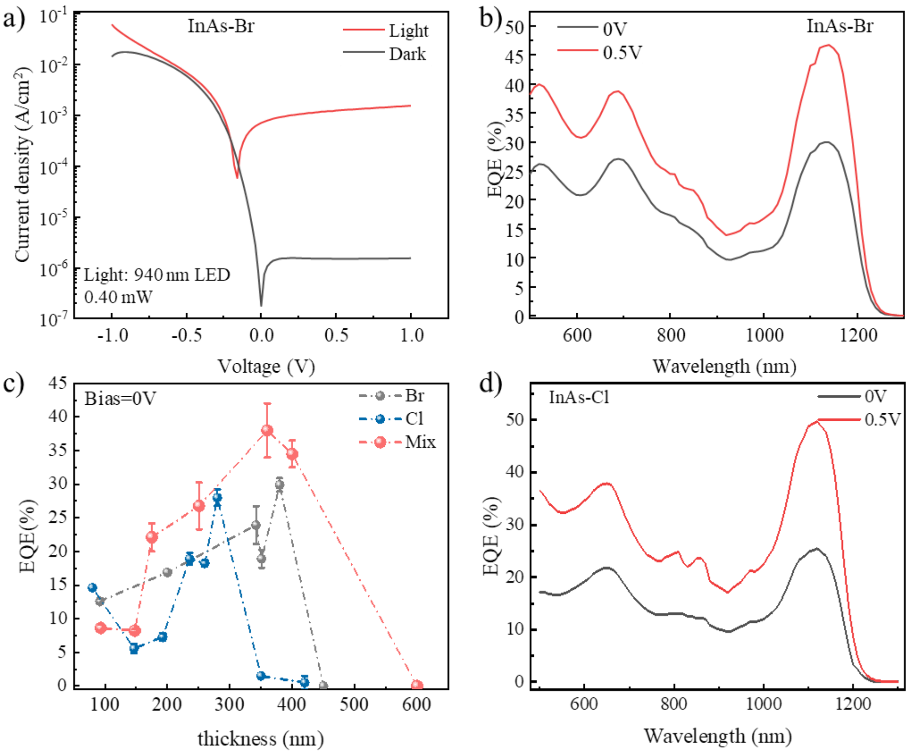

Figure S12. Device performance. A)current density−voltage and the b) EQE of Br−passivated InAs CQD based-photodetectors.c) average thickness dependence of the EQE at 0 voltage. The film thickness is measured by profilometer, and the optimized thickness has been calculated with more than 16 devices to give a standard error. d) a typical EQE of InAs-Cl photodetector with optimized thickness.

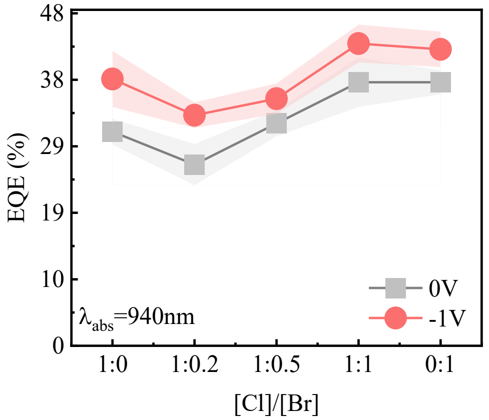

Figure S13. EQE of Br-, mixed Br/Cl-, and Cl-passivated 940 nm InAs CQD photodetectors. The devices are fabricated in an NIP structure, as reported in our previous study. [1]

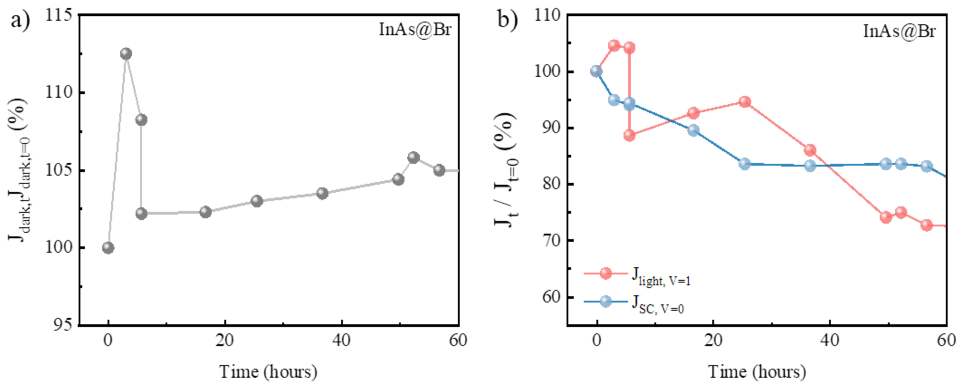

Figure S14. Dark current stability (a) and light current stability (b) of the InAs-Br QD-based photodetectors under illumination.

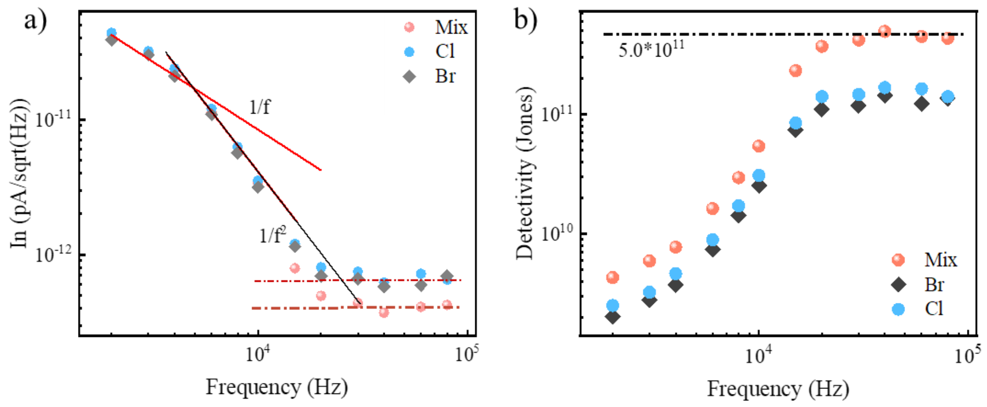

Figure S15. Noise measurement a) and the calculated detectivity b) of Br, Mix and Cl passivated InAs CQDs.

Table S4. Statistics of the InAs QD based photodiode-type photodetectors.

NO	Active layer	Exciton peaks/nm	Bias/V	EQE/%	Dark current mA/cm^2	Detectivity /Jones	ref
1	InAs QD	940	0	30	8.00E-08	1.00E+11	[2]
			1	40			
2	InAs QD	940	0.1	18	5.00E-07	2.00E+11	[3]
			1	36			
3	InAs QD	940	0	37	3.00E-08	1.90E+11	[1]
			1	40			
4	InAs QD	940	0	40	4.00E-07	6.90E+11	[4]
			1	53			
			1	75	2.00E-07		
5	InAs QD	1130	0	30	2.00E-07	-	[5]
			1	35			

6	InAs QD	1500	0	-	1.00E-05	1.20E+10	[6]
			1	15			
7	InAs(P)-QD	1150	-1	0.3	2.00E-06	1.10E+10	[7]
			-4	5			
		1270	-4	5	-	6.10E+09	
		1420	-4	1	-	1.00E+09	
**8**	**InAs QD**	**1140**	**0**	**40**	**2.00E-07**	**5.00E+11**	**This work**
			**1**	**75**			

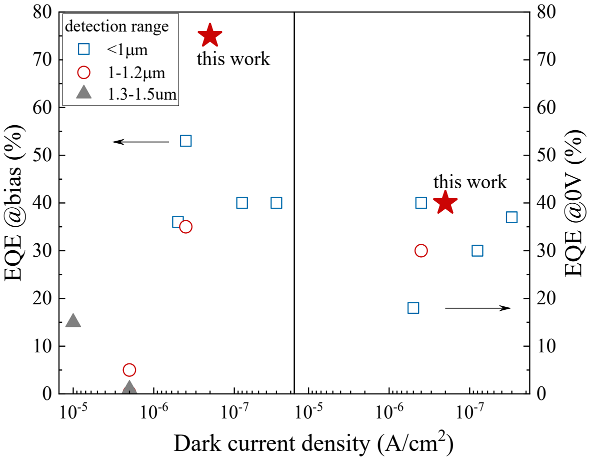

Figure S16. EQE and dark current density (@1V) of InAs CQD photodetectors with and without bias. Reference data from Table S4.

Experimental sections

Computational methods

Density functional theory (DFT) calculations were performed using the projector-augmented wave method [8]using the Vienna Ab initio Simulation Package (VASP) [9,10]. The strongly constrained and appropriately normed (SCAN) functional was implemented to describe the exchange-correlation interactions[11,12]. A kinetic cutoff energy of 400 eV was employed for all calculations. The van der Waals interactions were included using the DFT-D3 method with Becke-Johnson damping [13,14]. All structures were fully relaxed until the residual force on each ion was smaller than 0.05 eV· $Å^{-1}$, and energy differences converged within 10^{-4} eV/atom. Charge-balanced InAs QD models were used with a diameter of approximately 3nm. The surface of initial InAs QD structures were fully passivated by Br or Cl ligands.

Colloidal Quantum Dot Synthesis

InAs QDs were synthesized using a modified approach via continuous injection by reacting $In(oleate)_3$, tris(trimethylsilane)arsine ($(TMSi)_3As$) under a nitrogen atmosphere at 300 °C using a modified approach.[15–17]

Two-Step InAs CQD Ligand Exchange

Br passivated CQDs: The ligand exchange follows previous work.[2] To briefly explain, 50 mg of InAs CQDs in 1 mL of octane was mixed with a 3 mL solution of $InBr_3$ and ABr, 0.02 mg in DMF

(dimethylformamide). CQDs were transferred to the DMF phase after a 2-minute vortex and then crashed out with acetone. Followed by another ligand exchange with the same condition as $InBr_3$ and ABr (ammonium bromide) in mixture of GBL (gamma-Butyrolactone) and DMSO (dimethyl sulfoxide).

Mixture-halides passivated CQDs followed similar recipes but use combination of $InBr_3$ and $InCl_3$ instead, the molar ratio has been modified to find the 1: 1 ratio of Br to Cl is the best. Cl passivated CQDs also follow the same steps but use $InCl_3$ to replace $InBr_3$ instead.

Colloidal Quantum Dot Photodiode Fabrication (NIP)

The NIP devices were fabricated following previous work.[1] The ZnO nanoparticles is fabricated and then were spin-cast on the ITO substrate at 3000 rpm for 20 s (≈20 nm thickness). The CQDs after ligand exchange were spin-cast on the ZnO/ITO substrate with a concentration of 200–300 mg/mL of CQD inks in a GBL: DMSO solution with dynamic spinning (2.5 k rpm) in a N_2-filled glovebox (≈150-300 nm thickness). 30 nm InAs with MCE ligands are obtained by following previous report[5] and then spin-coating on InAs CQD film, followed by 14 nm MoO_X and 140 nm Ag as the top electrode, which was deposited by thermal evaporation. Note: different batches of InAs CQD have been tested to repeat the results, as the absorption ranging from 1110- 1140 nm. The thickness of film is measured with a Bruker Dektak profilometer.

Colloidal Quantum Dot Photodiode Fabrication (PIN)

The PIN devices are fabricated following previous report but using InAs CQD with 180-220 nm thickness range.[4]

Current-voltage and External Quantum Efficiency Measurement

Current-voltage characteristics were measured with a Keithley 2400 source in dark conditions. Devices were tested under continuous nitrogen flow. The I-V curves were scanned from -1.0 to +1.0 V at 0.02 V interval steps. EQE spectra were measured using an Enlitech QE-R Quantum Efficiency Analyzer (400–1700 nm, 10 nm resolution) with mechanically chopped monochromatic light at 210 Hz. The output power was calibrated using silicon and germanium detectors.

TPC and photodetector stability measurement

Both measurements were conducted as reported in previous works.[1,2] The photodetector's temporal response was measured using a 1-GHz Tektronix oscilloscope and an 850 nm VCSEL modulated at 20 kHz via a Tektronix AFG31000 function generator. The signal was preamplified (10^3 V/A) with a Femto DHPCA-100 preamplifier, which also provided bias when needed. Stability testing is conducted under a continuous LED light (MCLS1-940) with 4.0 mW/cm^2. Here the InAs-mixture based photodetectors applied in stability measurement are fabricated with PIN structure to avoid the effect of ZnO NP as previous reported.[1,4] The PIN structure based PbS CQD photodetectors are fabricated by following previous work with 75% EQE at 0 voltage.[18,19]

Noise-equivalent power measurement and specific detectivity calculation

Noise spectra were measured using a lock-in amplifier (SR 830) combined with a SR570 low-noise pre-amplifier. Noise measurements were carried out in the dark at room temperature for the pixels with a 0.1 cm^2 area. The noise current was calculated using the noise signal measured at 20 kHz and a sensitivity of of 10^7 V/A from the preamplifier. The detectivity was calculated based on noise current, responsivity, a set bandwidth of 1 Hz, and an active area of 0.1cm^2,[18,20] by following the recipes from previous work.[1]

Absorption, PL and TRPL measurement

Absorption of solution and thin film are measured with PerkinElmer Lambda 900 UV/VIS/NIR Spectrometer. The PL are measured with Horiba with 450 nm excitation. To acquire transient PL, a time-correlated single photon counting detector and a pulse near infrared laser diode (DD-830L, DeltaDiode, 820 nm, HORIBA Scientific) were used. The sample was illuminated 15° with respect to the surface normal. The photoluminescence was collected at 75° with respect to the surface normal and measured with a iHR320 spectrometer (HORIBA Scientific, 600 grooves mm−1, 1000 nm blaze wavelength) and InGaAs photodetector (S1-H10330-75). Lifetime of TRPL is fitted with equation S1

$$f(x) = \sum_{n}^{1,2,3}(A_i * \exp\left(-\frac{t}{t_i}\right)) \qquad \text{S1.}$$

FET fabrication and measurement

The field effect transistor substrate was fabricated with photolithography method by applying UV-resin to pattern the electrodes on p^{++}doped 291 nm thermal oxides silicon wafers. The specs of the used transistor have following parameters, channel width W = 100 μm, channel length L = 2 μm, n = 625 (number of fingers), thus W/L aspect ratio = 62450 (Calculated by n*W/L due to IDE structure). The transfer curves are measured with the applied drain-source voltage, V_{DS} =-30V and the output curves are measured with varying the gate voltage, V_G, and scan the V_{DS} voltage. Hole mobility is calculated by using data of linear regime ($V_{DS} << V_G - V_{th}$) from the transfer curves with equation S2.

$$\mu = (\frac{\partial I_{DS}}{\partial V_{GS}})/(C_{dielectric} \times V_{DS}) \times L/W \qquad \text{S2.}$$

Here $C_{dielectric}$ is the gate capacitance per unit area. Due to non-ideal of the CQD FET, two $\frac{\partial I_{DS}}{\partial V_{GS}}$ is used here, one as average mobility and one as maximum mobility. Hysteresis is calculated by the integration area J-V ratio of scanning back and forward, which indicates holes traps during one scan.

Space-charge-limited current (SCLC) measurement

Hole measurements are measured by fabricated hole only devices (ITO/ NiOx nanoparticle (20 nm) /InAs CQD-different ligands (250nm)/MoO_X (10nm)/Ag) to get the J-V curves and thus applied the equation to get the trap density from trap filling regime and the mobility following by using equation S3 and S4. [1]

$$J=9/8*\varepsilon\varepsilon_0\mu/(V_{app}-V_{bi})^2/L^3 \qquad \text{S3.}$$

where J is the current density in the SCLC region, V_{app} is the applied voltage, V_{bi} is the built-in voltage, ε_0 is the permittivity of free space (8.854×10^{-12} F/m), ε_r is the relative dielectric constant of the thin film μ is the charge carrier mobility, and L is the thickness of the thin film which is measured by profilometer.

The trap density can be extracted from the plot of the J-V curve on a log–log scale and calculate the trap density from the so-called trap-filled-limit voltage:

$$N_t=2V_{TFL}\varepsilon_r\varepsilon_0/eL^2 \qquad \text{S4.}$$

where V_{TFL} is the trap-filled limit voltage and e is the electron charge and n_t the trap density.

X-ray Photoelectron Spectroscopy (XPS)

XPS measurements were carried out on a Thermofisher Scientific K-Alpha spectrometer using Al Kα radiation (1486.6 eV) for excitation. The CQD films were prepared by spin-coating on ITO substrates. The air exposure of film is conducted by exposing samples to air with 55% humidity.

Ultraviolet photoelectron spectroscopy (UPS) measurement

A helium discharge source (HeI α, hV = 21.22 eV) was used, and the samples were kept at a take-off angle of 88°. During measurement, the sample was held at a -5 V bias relative to the spectrometer to collect low-kinetic-energy electrons efficiently. EF was calculated from the equation: EF = 21.22 eV - S_{EC}, where S_{EC} is the secondary electron cutoff. The difference between the valence band (VB) and EF, η, was determined from the VB onset in the VB region.

Transmission Electron Microscopy (TEM) Characterization

Bright-field TEM, high-resolution TEM, and STEM-HAADF images of the QD samples were acquired using a Hitachi HF-3300 transmission electron microscope operating at an accelerating voltage of 300 kV. The samples were prepared by drop casting diluted solutions of NCs onto carbon-coated 200 mesh copper grids. The characterization was performed by using a JEOL JEM2200 image-corrected instrument operated at 200 kV. Partial high-resolution transmission electron microscopy (HRTEM) and EDX characterization were taken on a Thermo Fisher Scientific Titan Cubed Themis G2 300 transmission electron microscope with an acceleration voltage of 300 kV and equipped with a Super-X4 probe super energy spectrum.

Powder X-ray scattering diffraction (PXRD)

XRD measurements were conducted using a Rigaku MiniFlex 600 diffractometer (Bragg-Brentano geometry) equipped with a NaI scintillation counter detector and a monochromatized Cu Kα radiation source (λ = 1.5406 Å) operating at a voltage of 40 kV and current of 15 mA.

Reference

[1] P. Xia, B. Sun, M. Biondi, J. Xu, O. Atan, M. Imran, H. Yasser, Y. Liu, J. Pina, A. Najarian, L. Grater, K. Bertens, L. K. Sagar, H. Anwar, M.-J. Choi, Y. Zhang, M. Hasham, F. P. G. de Arquer, S. Hoogland, M. W. B. Wilson, E. H. Sargent, *Adv. Mater.* **2023**, *35*, 2301842.

[2] B. Sun, A. M. Najarian, L. K. Sagar, M. Biondi, M.-J. Choi, X. Li, L. Levina, S.-W. Baek, C. Zheng, S. Lee, A. R. Kirmani, R. Sabatini, J. Abed, M. Liu, M. Vafaie, P. Li, L. J. Richter, O. Voznyy, M. Chekini, Z.-H. Lu, F. P. García de Arquer, E. H. Sargent, *Adv. Mater.* **2022**, *34*, 2203039.

[3] M.-J. Choi, L. K. Sagar, B. Sun, M. Biondi, S. Lee, A. M. Najjariyan, L. Levina, F. P. García de Arquer, E. H. Sargent, *Nano Lett.* **2021**, acs.nanolett.1c01286.

[4] P. Xia, T. Zhu, M. Imran, J. M. Pina, O. Atan, A. M. Najarian, H. Chen, Y. Zhang, E. Jung, M. Biondi, M. Vafaie, C. Li, L. Grater, A. Khatri, A. Singh, S. Hoogland, E. H. Sargent, *Adv. Mater.* **2024**, *36*, 2310122.

[5] M. Vafaie, A. Morteza Najarian, J. Xu, L. J. Richter, R. Li, Y. Zhang, M. Imran, P. Xia, H. W. Ban, L. Levina, A. Singh, J. Meitzner, A. G. Pattantyus-Abraham, F. P. García de Arquer, E. H. Sargent, *Proc. Natl. Acad. Sci.* **2023**, *120*, e2305327120.

[6] T. Sheikh, W. J. Mir, A. Alofi, M. Skoroterski, R. Zhou, S. Nematulloev, M. N. Hedhili, M. B. Hassine, M. S. Khan, K. E. Yorov, B. E. Hasanov, H. Liao, Y. Yang, A. Shamim, M. Abulikemu, O. F. Mohammed, O. M. Bakr, *J. Am. Chem. Soc.* **2024**, *146*, 29094.

[7] J. Leemans, V. Pejović, E. Georgitzikis, M. Minjauw, A. B. Siddik, Y.-H. Deng, Y. Kuang, G. Roelkens, C. Detavernier, I. Lieberman, P. E. Malinowski, D. Cheyns, Z. Hens, *Adv. Sci.* **2022**, *9*, 2200844.

[8] P. E. Blöchl, *Phys. Rev. B* **1994**, *50*, 17953.

[9] G. Kresse, *Phys. Rev. B* **1996**, *54*, 11169.

[10] G. Kresse, *Phys. Rev. B* **1999**, *59*, 1758.

[11] J. P. Perdew, *Phys. Rev. B* **1992**, *46*, 6671.

[12] J. Sun, *Phys. Rev. Lett.* **2015**, *115*, DOI 10.1103/PhysRevLett.115.036402.

[13] S. Grimme, S. Ehrlich, L. Goerigk, *J. Comput. Chem.* **2011**, *32*, 1456.
[14] S. Grimme, J. Antony, S. Ehrlich, H. Krieg, *J. Chem. Phys.* **2010**, *132*, 154104.
[15] D. Franke, D. K. Harris, O. Chen, O. T. Bruns, J. A. Carr, M. W. B. Wilson, M. G. Bawendi, *Nat. Commun.* **2016**, *7*, 12749.
[16] T. Kim, S. Park, S. Jeong, *Nat. Commun.* **2021**, *12*, 3013.
[17] L. K. Sagar, G. Bappi, A. Johnston, B. Chen, P. Todorović, L. Levina, M. I. Saidaminov, F. P. García de Arquer, D.-H. Nam, M.-J. Choi, S. Hoogland, O. Voznyy, E. H. Sargent, *Chem. Mater.* **2020**, *32*, 7703.
[18] M. Vafaie, J. Z. Fan, A. Morteza Najarian, O. Ouellette, L. K. Sagar, K. Bertens, B. Sun, F. P. García de Arquer, E. H. Sargent, *Matter* **2021**, *4*, 1042.
[19] O. Atan, J. M. Pina, D. H. Parmar, P. Xia, Y. Zhang, A. Gulsaran, E. D. Jung, D. Choi, M. Imran, M. Yavuz, S. Hoogland, E. H. Sargent, *Nano Lett.* **2023**, *23*, 4298.
[20] M. Biondi, M. Choi, Z. Wang, M. Wei, S. Lee, H. Choubisa, L. K. Sagar, B. Sun, S. Baek, B. Chen, P. Todorović, A. M. Najarian, A. Sedighian Rasouli, D. Nam, M. Vafaie, Y. C. Li, K. Bertens, S. Hoogland, O. Voznyy, F. P. García de Arquer, E. H. Sargent, *Adv. Mater.* **2021**, 2101056.
